# Compositions and Antioxidant Activity of Tea Polysaccharides Extracted from Different Tea (*Camellia sinensis* L.) Varieties

**DOI:** 10.3390/foods12193584

**Published:** 2023-09-27

**Authors:** Kunyue Xiao, Yutao Shi, Sisi Liu, Yuqiong Chen, Dejiang Ni, Zhi Yu

**Affiliations:** 1 College of Horticulture & Forestry Sciences, Huazhong Agricultural University, Wuhan 430070, China; xky@webmail.hzau.edu.cn (K.X.); ykmoon22@163.com (S.L.); chenyq@mail.hzau.edu.cn (Y.C.); nidj@mail.hzau.edu.cn (D.N.); 2 College of Tea and Food Sciences, Wuyi University, Wuyishan 354300, China; ytshi@wuyiu.edu.cn; 3 Tea Engineering Research Center of Fujian Higher Education, Wuyishan 354300, China

**Keywords:** tea varieties, tea polysaccharides, composition, bioactivity

## Abstract

Tea polysaccharide (TPS) is a bioactive compound extracted from tea. It has raised great interest among researchers due to its bioactivity. However, few studies focused on the diversity of TPS in its compositions and antioxidant activity. This study collected 140 different tea varieties from four tea germplasm gardens in China, and their TPSs in tea shoots were extracted. The extraction efficiency, composition contents, including neutral sugar, uronic acid, protein, and tea polyphenols, and the scavenging abilities of hydroxyl radical (·OH) and superoxide radical (O2-·) of 140 TPSs were determined and analyzed. The results showed significant differences in the compositions and antioxidant activities of TPS extracted from different tea varieties. By applying hierarchical clustering analysis (HCA), we selected nine tea varieties with high TPS extraction efficiency and 26 kinds of TPS with high antioxidant capacity.

## 1. Introduction

Tea (*Camellia sinensis* L.) is the second most popular beverage after water and is loved by consumers worldwide for its exceptional flavor and healthy functions [1,2]. Studies have demonstrated the health benefits of tea, including hyperglycemic activity, antioxidant properties, hepatoprotective, immunoregulatory, antiviral, antitumor, and anti-obesity, which are mainly attributed to theanine, tea polyphenols, flavonoids, and tea polysaccharides (TPS) [3,4]. TPS is an acidic glycoprotein substance extracted from tea [5]. TPS possesses various bioactivities, including hypoglycemic activity, antidiabetic, antitumor, and antioxidant, which have been widely utilized in the food and medical industry [6,7,8,9].

TPS is a complex glycoprotein compound comprising multiple compositions, mainly neutral sugar, uronic acid, and protein [10]. It has been reported that TPS possessed a positive effect on scavenging reactive oxygen species (ROS), including hydroxyl radical (·OH) and superoxide radical (O2-·) [11,12,13]. The compositions and antioxidant activity of TPS vary with different forms of tea. Previous research has studied TPS isolated from green tea (GTPS), black tea (BTPS), and oolong tea (OTPS) and discovered that the protein content in BTPS was higher than that of GTPS and OTPS. In contrast, the uronic acid and neutral sugar contents were lower than those of GTPS and OTPS. Additionally, it revealed that the free radical scavenging activity of GTPS and BTPS was superior to that of OTPS [14]. However, most studies focused on analyzing TPSs extracted from commercial tea [15,16,17]. Very few publications discussed the differences in composition and antioxidant capacity of TPS extracted from shoots of various tea varieties.

Tea plants are grown worldwide with giant species, making TPS a broad developing prospect [18]. TPS varies with tea cultivars [19]. Fibers of TPS are incorporated in cuticular membranes (CM) [20]. Tsubaki et al. found that the tea variety “Yabukia” with thicker CM had a higher TPS yield compared to “Samidori” and “Gokou” with lighter CM [21]. Plants can be successfully engineered as bio-factories for synthesizing biomolecules such as phenolic compounds [22]. For tea plants, it is feasible to isolate TPSs from the tea shoots and construct a “TPS factory” for synthesizing TPS-rich products such as healthcare products. The “TPS factory” production relies on the quality and quantity of raw TPS as its foundation. Therefore, efforts should be focused on enhancing TPS extraction technology and searching for raw tea materials containing a high TPS volume. Researchers have been focused on optimizing the TPS extraction method [23,24,25]. However, systematic studies on TPSs’ composition and antioxidant activity in tea cultivars have not been reported. 

The objectives of this study were to investigate the compositions and antioxidant activity of TPSs extracted from leaves of various tea varieties and to select tea varieties with abundant TPS content. Therefore, 140 tea shoots of different *Camellia sinensis* L. varieties were collected, and their TPSs were obtained and analyzed. Analysis of variance (ANOVA) and hierarchical clustering analysis (HCA) were utilized in this study. The results showed that TPSs’ compositions and bioactivities varied with different tea species. The HCA results identified tea varieties of high TPS content and TPSs with potent antioxidant capacity. 

## 2. Materials and Methods

### 2.1. Chemicals and Reagents

Anthrone-sulfuric acid, glucose, sulfuric acid, sodium tetraborate, carbazole, ferric tartrate, sodium dihydrogen phosphate, dipotassium hydrogen phosphate, potassium hydroxide, diamine tetraacetic acid, ferric chloride, hydrogen peroxide, ascorbic acid, hydrochloric acid, 95% ethanol, and coomasylan bright blue (CBB) G-250 were purchased from Sinopharm Group Chemical Reagents Co. (Shanghai, China). The superoxide dismutase (SOD) test kit was purchased from Jiancheng Biological Engineering Institute (Nanjing, China). The bovine serum protein was purchased from Wuhan Tianyuan Biotechnology Co. (Wuhan, China). D-ribose was purchased from Sigma-Aldrich (Beijing, China).

### 2.2. Tea Samples

In total, 140 tea (*Camellia sinensis* L.) varieties were collected in the same summer in July to August from four institutes in China, including Jiangxi Province Sericulture and Tea Research Institute in province (Jiangxi), Yunnan Province Academy of Agricultural Science Institute of Tea (Yunnan), Chongqing Academy of Agricultural Science (Chongqing), and Huazhong Agricultural University in Hubei province (Hubei). The tea varieties’ names and abbreviations can be seen in the Supplementary Materials (Table S2). Their fresh tea shoots of one bud and two leaves were plucked individually, and 140 different tea shoots were obtained. The fresh shoots were then immediately de-enzymed by steaming for 5 min. Subsequently, the steamed leaves were dried at 95 °C in a drying machine until the moisture was about 5%. These 140 tea samples were obtained. The tea samples were re-dried at 80℃ for 1 h before experiments.

### 2.3. TPS Extraction

TPSs in tea samples were extracted using the previously described method [14]. First, the tea samples were crushed into powders by grinder (FZ102, Qi Household Science Instrument Factory of Huanghua City, Hebei province, China). Next, 10.0 g of tea powder was extracted for 2.5 h using distilled water (200 mL) at 55 °C. The extracted substance was centrifuged (LD5-10, Beijing Medical Centrifuge Factory Co., Beijing, China) at 4000 r/min for 15 min. Subsequently, the supernatant was precipitated with 95% ethanol. Finally, the extracts were freeze-dried by a vacuum freeze dryer (ALpHAL-2, Martin Christ Freeze Dryers, Saxony, German), and TPSs were obtained. The TPS extraction efficiency (%) was the TPS weight accounting for the total weight of the dry tea sample.

### 2.4. Neutral Sugar Content Determination

The literature method was applied to determine the neutral sugar content of TPS with some modifications [26]. The prepared TPS aqueous solution (0.5 mL, 1 mg/mL) was mixed with anthrone-sulfuric acid (5.0 mL). Then, the mixture was incubated in a water bath at 100 °C for 7 min. The reaction mixture was allowed to cool and shake well. Finally, the absorbance of the cooled mixture was measured by an ultraviolet-visible (UV-VIS) spectrophotometer (CARY 50Scan, Varian Medical Systems Inc., California, USA) at a wavelength of 620 nm. Glucose was used as a reference standard. The neutral sugar content was then calculated from the glucose calibration curve. 

### 2.5. Uronic Acid Content Determination

The sulfuric acid-carbazole method was used to measure the uronic acid content of TPS [27]. The TPS solution (0.2 mL, 1.0 mg/mL) was gradually added with sulfuric acid (5.0 mL) containing sodium tetraborate (0.025 mol/L). Then, the mixture was incubated for 10 min at 100 °C. Finally, carbazole (0.2 mL, 0.125%, *w*/*v*) was added into the mix for further heating at 100 °C for 15 min. The UV-VIS spectrophotometer obtained the resulting solution’s absorbance at 530 nm.

### 2.6. Protein Content Determination

CBB G-250 was used for protein content detection. After binding with the protein, the color of CBB G-250 turns red to cyan, and the protein pigment shows maximum absorption at 595 nm. The protein content of TPS was evaluated according to the method reported by Bradford [28]. In brief, a TPS aqueous solution (0.5 mL, 1 mg/mL) was prepared and completely reacted with CBB G-250 (3.0 mL) for 10 min at room temperature. Finally, the absorbance was obtained at a wavelength of 595 nm. The protein content was then calculated from the bovine serum protein calibration curve.

### 2.7. Tea Polyphenols Content Determination

The tea polyphenols (TP) content of TPS was determined by the ferric tartrate colorimetric method with some modifications [29]. The distilled water (4.0 mL) and ferric tartrate (5.0 mL, 5.0 mg/mL) were added to the TPS solution (1.0 mL, 5.0 mg/mL). Then the mixture was added with the phosphoric acid buffer (15.0 mL, pH 7.5), which was prepared with sodium dihydrogen phosphate (1/15 mol/L) and dipotassium hydrogen phosphate (1/15 mol/L) at a ratio of 17:3 (*v*/*v*). The reaction was set at room temperature for 10 min. Finally, the absorbance was measured at a wavelength of 540 nm. The TP content was then calculated using the following equation:

TP (%) = E × 2 × 1.957/C × V × 100 (1)

where E represented the absorbance of the solution, 1.957 represented that the solution contained 1.957 mg of TP when the absorbance value was 0.5, and C and V were the solution’s concentration and volume, respectively.

### 2.8. Determination of O2-· Scavenging Ability 

TPSs’ O2-· scavenging ability was evaluated using a SOD test kit. A working solution of TPS at a concentration of 1.0 mg/mL was prepared for the test. The procedure was constructed following the instructions. The relative O2-· scavenging efficiency was determined by measuring the SOD activity (nU/mL).

### 2.9. Determination of ·OH Scavenging Ability

According to the literature [11], the ·OH scavenging ability was determined as follows. First, 0.1 mL of TPS aqueous solution (5.0 mg/mL) was sequentially added with dipotassium hydrogen phosphate (KH_2_PO_4_/KOH) buffer (0.4 mL, 50 mmol/L), ethylene diamine tetraacetic acid (0.1 mL, 1.04 mmol/L), and ferric chloride (0.1 mL, 1 mmol/L). Next, the above mixture was fully mixed with hydrogen peroxide (0.1 mL, 10 mmol/L), D-ribose (0.1 mL, 60 mmol/L), and ascorbic acid (0.1 mL, 2.0 mmol/L) and incubated at 37 °C for 1 h. After that, hydrochloric acid (1.0 mL, 25%, *v*/*v*) was used to terminate the reaction. Finally, thiobarbituric acid (1.0 mL, 1%, *w*/*v*) was added, and the reaction was conducted for 15 min in the boiling water bath. After cooling, the absorption was measured at 532 nm. A control experiment was performed using distilled water. The ·OH scavenging rate was calculated according to the following formula:

·OH scavenging ability (%) = (A_0_ − (A_1_ − A_2_))/A_0_ × 100 (2)

A_0_, A_1_, and A_2_ represented the absorbance of the solution without D-ribose, the solution with D-ribose, and the control experiment, respectively.

### 2.10. Statistical Analysis

The contents of all components and scavenging ability were detected in triple, and the mean value was taken to represent the result. Microsoft Excel and Origin Software version 2021 were used in the statistical treatment of the data. For a parametric distribution, one-way analysis of variance (ANOVA) and least significant difference (LSD) test were used to analyze the statistical significance. The R studio software (Version 2022.02) was used for hierarchical clustering analysis (HCA) and data visualization.

## 3. Results

### 3.1. TPS Extraction Efficiency of Different Tea Varieties

**Figure 1:**
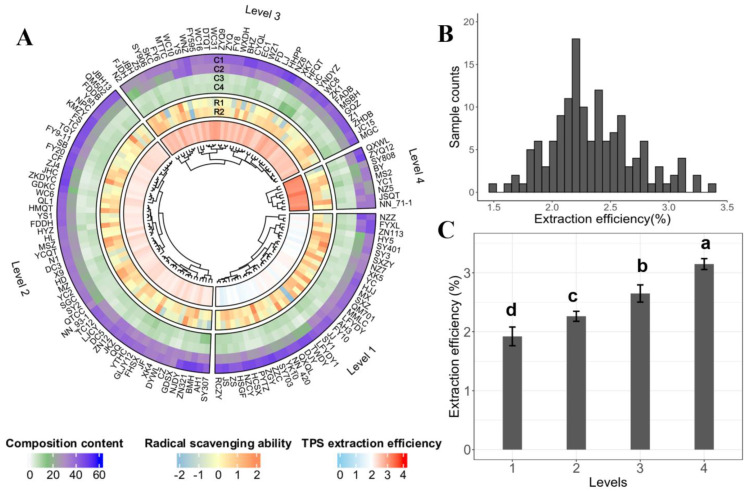
Differences in extraction efficiency, composition content, and radical scavenging ability of TPSs from 140 tea varieties. (**A**) Complex heatmap of different components content and antioxidant capacity of 140 TPSs. The inner track (blue−red−colored track) is the hierarchical clustering result of the TPS extraction efficiency. The intermediate track (blue−orange−colored track) is the radical scavenging capacity of TPS. The external track is the distribution of four components of TPS. C1−C4: neutral sugar, uronic acid, protein, and tea polyphenols. R1–R2: hydroxyl radical, and superoxide radical. (**B**) Distribution of TPS extraction efficiency among tea varieties. (**C**) Four levels of TPS extraction efficiency. Different letters significantly differ by the LSD test (*p* < 0.01).

The TPS content in tea samples varies with raw material, manufacturing process, and extraction methods. Here, to minimize the effect of processing technology on TPS, tea samples were prepared with a simple procedure of steaming and drying. As shown in Figure 1A, the extracted efficiency of TPS from 140 samples differed. The TPS extraction efficiency of most tea varieties ranged from 2% to 3%, and only a few species possessed lower or higher TPS content (Figure 1B). Among the 140 species, MS2 had the maximum TPS content (3.34%), while JS demonstrated the minimum TPS content (1.46%). The variety with the highest TPS extraction efficiency was over twice that with the lowest, indicating a noticeable variety difference among the TPS extraction efficiency. Hierarchical clustering analysis (HCA) is a typical data measurement for finding the samples with maximum similarities from numerous sample groups. In this study, HCA was performed on the TPS extraction efficiency of 140 tea varieties to identify those varieties with higher TPS yield. It can be seen that these 140 varieties were clustered into four major clusters by the TPS extraction efficiency, namely Level 1, Level 2, Level 3, and Level 4. The average TPS extraction efficiency of Level 4 was significantly higher than that of the other three levels. Tea varieties classified in Level 4 exhibited TPS extraction efficiency surpassing 3.0%. Notably, the tea varieties of MS2 (3.34%), QXWL (3.22%), NN_71-1 (3.21%), YC1 (3.13%), ZYQ12 (3.12%), NZ5 (3.11%), SY808 (3.10%), BY (3.07%), and JSQT (3.04%) could stand out as potential high TPS content tea resources. 

### 3.2. Composition Contents of TPS

In total, 140 TPSs were analyzed to determine the contents of neutral sugar, uronic acid, protein, and tea polyphenols. The results were graphically represented in Figure 1A (green-purple-colored track). The composition content was observed in the following order: neutral sugar > uronic acid > protein > tea polyphenols. Table 1 displays the variation of these four compositions across the 140 TPSs. As can be seen, the content of neutral sugar, uronic acid, protein, and tea polyphenols ranged from 23.82% to 49.13%, 13.54% to 45.38%, 1.76% to 9.21%, and 2.45% to 16.12%, respectively. Neutral sugar was identified as the most prominent compound in TPS. The maximum neutral sugar in TPS existed in the following four species: ZZC (49.19%), BMH (49.19%), NZ6 (49.11%), and ZN321 (49.03%). In comparison, YC1 had the lowest neutral sugar (23.81%), less than half the value of ZZC. The uronic acid content in tea polysaccharides ranked second only to neutral sugar (Table 1). TPS of NZZ exhibited the most uronic acid content of 45.38%, while TPS of N2 was observed with the lowest uronic acid content of only 13.54%. As for tea polyphenols, TPS of XK7 (16.12%), JC (14.46%), SY307 (13.20%), and HFQT (13.19%) contained higher levels compared to other varieties. The coefficient of variation for the four components across the 140 TPSs ranged from 14.26% to 30.60% in the following order: tea polyphenols (30.60%) > protein (28.79%) > uronic acid (20.75) > neutral sugar (14.26%). ANOVA analysis revealed that 140 TPSs demonstrated significant differences in neutral sugar, uronic acid, protein, and tea polyphenol contents (Table 2). These findings highlight the significant variation in these four components across different TPS varieties.

### 3.3. Radical Scavenging Abilities of TPS

The radical scavenging abilities of TPSs extracted from different tea varieties on hydroxyl radical (·OH) and superoxide radical (O2-·) were determined, and the results are visualized in Figure 1A. It can be seen that the radical scavenging capacity of TPSs demonstrated differences among tea species. The ·OH scavenging activity of TPSs ranged from 76.57% to 56.67%. TPS of ZZC had the maximum ·OH scavenging ability, while TPS of YS had the minimum ·OH scavenging capacity. The range of scavenging capacity of O2-· was from 4.26 nU/mL to 30.37 nU/mL. TPS extracted from the variety of SY1 and SXZY exhibited the highest and lowest O2-· scavenging ability, respectively. TPS of some varieties, such as JSQT, MTTC, ZJ4, YCQT, ZZC, DJY, and YC, exhibited excellent scavenging ability towards ·OH and O2-·. In contrast, the TPS of QXWL, EC1, SKC, SY906, and SY1 showed poor performance regarding the scavenging ability of these two radicals. The ANOVA analysis showed a significant difference in TPSs’ scavenging capacity towards ·OH and O2-· among the 140 tea varieties (Table 2).

### 3.4. Correlation Coefficient Analysis

**Figure 2:**
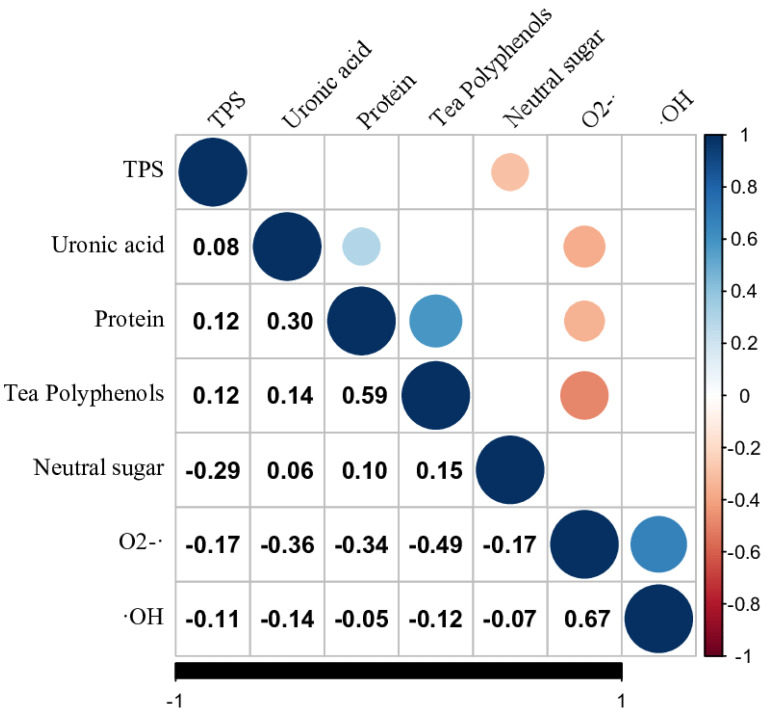
Correlation analysis on the extracted efficiency, composition content, and the hydroxyl radical (·OH) and superoxide radical (O2-·) scavenging capacity of TPS by Pearson correlation coefficient. Colored circles represent significance at *p* < 0.05.

A correlation analysis investigated the relationship between TPSs’ compositions and their radical scavenging activity. The content of neutral sugar, uronic acid, protein, and tea polyphenols was examined, along with the hydroxyl radical (·OH) and superoxide radical (O2-·) scavenging ability. As shown in Figure 2, the correlation between the content of neutral sugar, uronic acid, protein, tea polyphenols, and ·OH scavenging ability was not statistically significant (*p* < 0.05). However, a significant correlation was found between the content of uronic acid, protein, polyphenols, and O2-· scavenging ability, respectively. Surprisingly, the correlation between tea polyphenols content of TPS and O2-· scavenging capacity was found to be significantly negative, inferring that an increase of tea polyphenols in TPS may reduce the antioxidant activity of TPS. Moreover, the protein content showed significantly positive correlations with polyphenols and uronic acid content. A significant positive correlation was also observed between the ·OH scavenging ability and O2-· scavenging ability.

### 3.5. Analysis of TPSs extracted from Tea Shoots Collected in Different Areas

In this experiment, six tea varieties, including FADB, FDDH, FY6, MZ, WNZ, and Ysh, were shared by Jiangxi, Yunnan, and Hubei. The contents of neutral sugar, uronic acid, protein, and tea polyphenols in these TPSs were determined and compared, and the results are presented in Table 3. The data showed significant differences in the TPSs’ uronic acid, tea polyphenols contents, and the total content of four compositions among tea varieties. However, no significant differences among tea varieties were observed in the TPSs’ protein and neutral sugar contents (Table 3). As shown in Table 3, there were significant differences in the antioxidant activity of TPSs among the six tea varieties. The strongest hydroxyl radical (·OH) and superoxide radical (O2-·) scavenging ability were found in TPS of Ysh, while the lowest antioxidant activities were observed in FDDH samples. There was no significant difference in the ·OH scavenging ability among TPS of FADB, FDDH, FY6, MZ, and WNZ. There was also no significant difference in TPS composition content and antioxidant activity among samples from different origins. The highest ·OH and O2-· scavenging ability was found in the samples from Yunnan and Hubei, respectively (Table 3).

### 3.6. Selecting TPSs with High Antioxidant Activity

**Figure 3:**
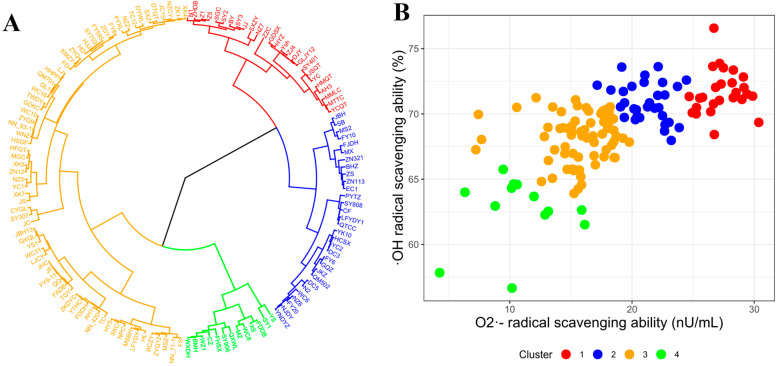
Cluster analysis of the antioxidant activity of 140 TPSs. (**A**) Cluster dendrogram of the antioxidant capacity of 140 TPSs. (**B**) Distribution of the free radical scavenging activity of 140 TPSs.

Hierarchical clustering analysis (HCA) was performed on the radicals scavenging abilities of 140 TPSs using standardized data as input. The results, shown in Figure 3A, revealed that the 140 TPSs could be categorized into four major clusters. The antioxidant activity of the TPSs followed a decreasing order: red cluster > blue cluster > orange cluster > green cluster (Figure 3B). Clusters 2 and 3 consisted of TPSs with a considerable scavenging capacity toward either hydroxyl radical (·OH) or superoxide radical (O2-·). TPSs in the green cluster demonstrated the weakest free radical scavenging ability. Conversely, TPSs in the red cluster (Cluster 4) exhibited the most potent antioxidant activities, displaying high scavenging capacity for both radicals. Cluster 4 consisted of 26 TPSs with high antioxidant activity, namely: N1, ZHDB, Z1, Z5, SY2, SGC, BY, JJ, SY3, NZ7, SXZY, ZZG, GDSX, HYZ, ZJ4, Ysh, DJY, GLJY12, SY401, YC, JSQT, AH3, HMQT, MMLC, YCQT, and MTTC. Additionally, the JSQT tea variety exhibited the highest TPS extraction efficiency (3.04%, Figure 1A), making it a suitable choice for producing TPS with antioxidant capacity. In summary, the HCA analysis effectively grouped the 140 TPSs based on their scavenging abilities, with TPSs in the red cluster demonstrating the highest antioxidant activity. The identified TPS sources in Cluster 4 and the JSQT hold promise for their potential use due to their high antioxidant activity.

## 4. Discussions

The processing technologies and different raw materials might affect the chemical composition and bioactivity variation of TPSs [10]. This study prepared the tea samples by steaming and drying to reduce the technical impact on TPS. Extraction with hot water or low-temperature water is a traditional method in the preparation of TPS [9]. Chen et al. extracted TPS from fresh tea leaves using distilled water at 90 °C for 2 h [30]. TPS from low-grade green tea was prepared using water with auxiliary enzymes at 40 °C for 4 h [12]. This study extracted 140 kinds of TPS from dried tea leaves using distilled water at 55 °C for 2.5 h. The TPS content was different between tea flowers (5.24%) and tea peel fruits (4.98%) [31,32]. The TPS yield was reported to be significantly different among green, black, oolong, white, yellow, and dark tea, ranging from 1.81% to 6.38% [33]. The present research found that the TPS yield varied from tea leaves of different tea varieties with the range of 1.40% to 3.34%.

TPSs’ chemical compositions also vary from the tea categories [33]. Xiang et al. found that there were significant differences in the protein, neutral sugar, and uronic acid content among BTPS, GTPS, OTPS, and tea polysaccharides of dark tea (DTPS) [34]. TPS derived from low-grade green tea possessed neutral sugar, uronic acid, and protein content of 37.03%, 14.09%, and 2.11% [12]. In this study, neutral sugar and uronic acid were identified as the predominant compositions, with an average content of 39.54% and 26.65%, respectively, which was consistent with the previous study [35]. The content of uronic acid, protein, and polyphenols strongly correlated with TPSs’ antioxidant ability, following the reported results [36,37]. Tea polyphenols were reported to be a non-toxic antioxidant with free radicals scavenging capacity [38,39]. Previous studies reported that tea polyphenols in tea products were positively correlated to antioxidant activity [37,38]. Wang et al. discovered that the content of polyphenols was relatively low in those TPSs with strong 2,2-diphenyl-1-picrylhydrazyl radical (DPPH) scavenging ability [40]. This study identified that tea polyphenols content was negatively correlated to the O2-· scavenging activity. In the present research, the O2-· scavenging activity was negatively correlated with the uronic acid content. In summary, the TPS’s antioxidant activity was related to its chemical composition.

## 5. Conclusions

Tea varieties with high TPS content can be the source of TPS extraction and can be built as a natural “TPS factory.” This study extracted TPSs from tea shoots of 140 different varieties. The extracted efficiency of TPS exhibited noticeable differences among the varieties, and nine tea species were identified as the high TPS content tea variety, including MS2, QXWL, BY, NN_71-1, YC1, ZYQ12, NZ5, SY808, and JSQT. The composition content, including neutral sugar, uronic acid, protein, and tea polyphenols, and the scavenging ability of hydroxyl radical (·OH) and superoxide radical (O2-·) of 140 TPSs were found to be significantly different, indicating the diversity of TPS varieties. The correlation analysis result showed a significant correlation between the antioxidant activity and the compositions of TPS. The scavenging ability of ·OH was positively correlated to the O2-· scavenging ability of TPS. In addition, 26 tea varieties were identified as potential TPS resources of high antioxidant capacity. Tea shoots of the JSQT variety had abundant TPS content, and its TPS exhibited excellent scavenging capacity towards ·OH and O2-·. The present research revealed the diversity of TPS among tea varieties and identified the tea varieties with high TPS content, and TPSs of superior antioxidant capacity were selected.

## Figures and Tables

**Table 1:** Variation of the compositions of TPS extracted from 140 tea varieties.

Composition	Content (%)				
	Max	Min	Mean	SD	CV
Neutral sugar	49.13	23.82	39.54	5.64	14.26
Uronic acid	45.38	13.54	26.65	5.53	20.75
Protein	9.21	1.76	5.21	1.5	28.79
Tea polyphenols	16.12	2.45	6.70	2.05	30.60

SD: standard deviation; CV: coefficient of variation.

**Table 2:** ANOVA analysis on composition content and radical scavenging capacity of TPS.

Analysis Items	SSM	SSE	F Value	*p*-Value
Neutral sugar content (%)	2.0172	0.0774	52.51	**
Uronic acid content (%)	1.6777	0.2734	12.36	**
Protein content (%)	0.7545	0.4362	3.48	**
Polyphenol content (%)	0.6330	0.0200	63.68	**
scavenging capacity of ·OH (%)	0.4570	0.3060	3.01	**
scavenging capacity of O2-· (nU/mL)	2.3528	0.2533	18.71	**

·OH: hydroxyl radical; O2-·: superoxide radical. SSM: sum square of the model; SSE: sum square of error; **: significant difference at *p* < 0.01.

**Table 3:** Comparison of components and antioxidant activity of TPS extracted from tea shoots collected in different areas.

TPS		Composition Content (%)					Scavenging Ability	
		Neutral Sugar	Protein	Uronic Acid	Tea Polyphenols	Total	·OH (%)	O2-· (nU/mL)
Varieties	FADB	34.72 ± 5.69 ^a^	5.87 ± 0.81 ^a^	26.06 ± 0.89 ^b^	6.75 ± 0.96 ^a^	73.98 ± 5.63 ^ab^	65.2 ± 3.73 ^b^	15.41 + 2.86 ^bc^
	FDDH	36.08 ± 4.09 ^a^	4.87 ± 1.22 ^a^	27.22 ± 2.38 ^ab^	6.03 ± 0.92 ^abc^	74.82 ± 4.18 ^ab^	62.77 ± 0.89 ^b^	11.37 + 0.66 ^c^
	FY6	33.00 ± 2.54 ^a^	4.45 ± 0.40 ^a^	24.43 ± 2.17 ^b^	6.59 ± 1.02 ^c^	67.34 ± 4.31 ^b^	67.38 ± 4.33 ^b^	16.06 + 2.13 ^bc^
	MZ	37.42 ± 4.94 ^a^	5.34 ± 1.13 ^a^	23.30 ± 4.79 ^b^	7.32 ± 0.48 ^ab^	73.13 ± 8.85 ^ab^	63.66 ± 2.45 ^b^	18.35 + 3.35 ^ab^
	WNZ	37.52 ± 3.68 ^a^	4.58 ± 1.61 ^a^	34.35 ± 6.71 ^a^	6.64 ± 0.54 ^bc^	82.29 ± 11.17 ^a^	66.69 ± 2.93 ^b^	15.18 + 2.83 ^bc^
	Ysh	36.11 ± 2.21 ^a^	4.85 ± 0.66 ^a^	26.29 ± 5.57 ^ab^	5.46 ± 0.65 ^abc^	73.64 ± 4.61 ^ab^	77.95 ± 3.69 ^a^	22.44 + 5.82 ^a^
Origins	Hubei	4.18 ± 4.18 ^a^	4.84 ± 1.58 ^a^	26.63 ± 2.90 ^a^	7.07 ± 0.81 ^a^	73.61 ± 3.94 ^a^	66.10 ± 4.71 ^a^	17.37 + 3.83 ^a^
	Jiangxi	3.77 ± 3.77 ^a^	4.88 ± 0.51 ^a^	26.00 ± 8.06 ^a^	5.85 ± 0.85 ^a^	72.25 ± 10.7 ^a^	66.33 ± 6.44 ^a^	16.46 + 6.44 ^a^
	Yunnan	3.89 ± 3.89 ^a^	5.26 ± 0.78 ^a^	28.2 ± 3.59 ^a^	6.39 ± 1.33 ^a^	76.74 ± 6.53 ^a^	69.40 ± 6.63 ^a^	15.57 + 3.03 ^a^

Different superscript letters denote significant differences (*p* < 0.05) by ANOVA analysis and LSD test.

## Data Availability

The data used to support the findings of this study can be made available by the corresponding author upon request.

## Supplementary Materials

The following supporting information can be downloaded at: https://www.mdpi.com/article/10.3390/foods12193584/s1, The abbreviations in this manuscript are listed in Table S1: Explanations of the abbreviations in the manuscript. Detailed information of the tea samples can be found in Table S2: Tea variety names and their abbreviations. Table S3. Turkey test and Dunn-Sidak test of the radical scavenging abilities of TPSs from different origins.
