# Supplementary material for: Compositions and Antioxidant Activity of Tea Polysaccharides Extracted from Different Tea (Camellia sinensis L.) Varieties
Source: Foods. 2023 Sep 27;12(19):3584. doi: 10.3390/foods12193584 (PMC10572641; doi:10.3390/foods12193584)
Supplement: Supplementary file 1 [file foods-12-03584-s001.zip › foods-2588156-supplementary.pdf]

Supplementary Materials

Compositions and Antioxidant Activity of Tea Polysaccharides Extracted from Different Tea (*Camellia sinensis* L.) Varieties

Kunyue Xiao[1,†], Yutao Shi[2,3,†], Sisi Liu[1], Yuchong Chen[1], Dejiang Ni[1], Zhi Yu[1,*]

[1] College of Horticulture & Forestry Sciences, Huazhong Agricultural University, Wuhan 430070, China; xky@webmail.hzau.edu.cn (K.X.); ykmoon22@163.com (S.L.); chenyq@mail.hzau.edu.cn (Y.C.); nidj@mail.hzau.edu.cn (D.N.)

[2] College of Tea and Food Sciences, Wuyi University, Wuyishan 354300, China; ytshi@wuyiu.edu.cn

[3] Tea Engineering Research Center of Fujian Higher Education, Wuyishan 354300, China

[*] Correspondence: yuzhi@mail.hzau.edu.cn

[†] The authors contributed equally to this paper.

Table S1. Explanations of the abbreviations in the manuscript.

Abbreviations	Explanations
·OH	Hydroxyl radical
ANOVA	Analysis of variance
BTPS	Tea polysaccharide of black tea
CBB	Coomasylan bright blue
DPPH	2,2-diphenyl-1-picrylhydrazyl radical
DTPS	Tea polysaccharide of dark tea
GTPS	Tea polysaccharide of green tea
HCA	Hierarchical clustering analysis
O2-·	Superoxide radical
OTPS	Tea polysaccharide of oolong tea
ROS	Scavenging reactive oxygen species
SOD	Superoxide dismutase
TP	Tea polyphenols
TPS	Tea polysaccharide
UV-VIS	Ultraviolet-visible

Table S2. Tea variety names and their abbreviations.

No.	Variety names	Abbreviations	No.	Variety names	Abbreviations
1	Zhuyeqi	ZYQ	71	Juhuachun	JHC
2	Zhuyeqi 9	ZYQ9	72	Huangmaquntizhong	HMQT
3	Nanjiangdaye	NJDY	73	Huangjinju	HJJ
4	Jiangsuqunti	JSQT	74	Zaoguanyin	ZGY
5	Wuniuzao	WNZ	75	Fuyunxilie	FYXL
6	Fujiandahong	FJDH	76	Dachun 5	DC5
7	Zhuyeqi 12	ZYQ12	77	Heichuanshuixian	HCSX
8	Jiukengzao	JKZ	78	Huangyezao	HYZ
9	Jingfeng	JF	79	Qinglin 1	QL1
10	Fuyun 10	FY10	80	Huangyou 5	HY5
11	Zhenghedabai	ZHDB	81	Dachun 3	DC3
12	Kunmingzhongye	KMZY	82	Longfengyandaye 1	LFYDY1
13	Foshou	FS	83	Zajiao 4	ZJ4
14	Jianbohuang 13	JBH13	84	Xikou 5	XK5
15	Fudingdahao	FDDH	85	Zhangkedayecui	ZKDYC
16	Baihaozao	BHZ	86	Ningzhouzhong	NZZ
17	Qianmei 502	QM502	87	Xikou 4	XK4
18	Zhenong 321	ZN321	88	Yichunqunti	YCQT
19	Niupicha	NPC	89	Maoxie	MX
20	Za 1	Z1	90	Anhui 3	AH3
21	Meizhan	MZ	91	Ningzhou 5	NZ5
22	Fudingdabai	FDDB	92	Guangdongshuixian	GDSX
23	Yinsun 1	YS1	93	Chunzao	CZ
24	Shukoucha	SKC	94	Wucha 10	WC10
25	Ningzhou 6	NZ6	95	Longfengyandaye	LFYDY
26	Fuyun 6	FY6	96	Shanxiziyang	SXZY
27	Gaoqiaozao	GQZ	97	Huangdan	HD
28	Longjingchangye	LJCY	98	Pingyangtezao	PYTZ
29	Fuandabai	FADB	99	Taicha 12	TC12
30	NN_93-1	NN_93_1	100	Tieguanyin	TGY

31	Fuyun 595	FY595	101	Echa 1	EC1
32	Fuyun 8	FY8	102	Yunnandayezhong	YNDYZ
33	Qiammei 701	QM701	103	Longjing	LJ
34	Jianbohuang	JBH	104	Yunkang 10	YK10
35	Dongtingqunti	DTQT	105	Mingshanzao	MSZ
36	Zhangke 1	ZK1	106	Shuyong 703	SY703
37	Fenghuangshuixian	FHSX	107	Shuyong 906	SY906
38	Magucha	MGC	108	Shuyong 401	SY401
39	Jinchun	JC	109	Shuyong 3	SY3
40	NN_420	NN_420	110	Yucha 1	YC1
41	Dajianye	DJY	111	Zao 5	Z5
42	Jiangcha 15	JC15	112	Mingshanbaihao	MSBH
43	Biyun	BY	113	Shuyong 2	SY2
44	Hefengqunti	HFQT	114	Quntichuancha	QTCC
45	Gudankucha	GDKC	115	Nan 2	N2
46	Xikou 7	XK7	116	Yucha 2	YC2
47	Meishan 2	MS2	117	Xuan 9	X9
48	Yingshuang	Ysh	118	Taiwandaye	TWDY
49	Anhui 1	AH1	119	Shuyong 808	SY808
50	Wucha 31	WC31	120	Nan 1	N1
51	Meitantaicha	MTTC	121	Qingxinwulong	QXWL
52	Tengcha	TC	122	Shuyong 1	SY1
53	Wucha 16	WC16	123	Zisun	ZS
54	Ruchengzaoya	RCZY	124	Chiyeqilan	CYQL
55	Wuxidahao	WXDH	125	Shuyong 307	SY307
56	Gelujiya 12	GLJY12	126	NN_71_1	NN_71_1
57	Wucha 8	WC8	127	Qilan	QQ
58	Yangtianhucha	YTHC	128	Fuding	FD
59	Shangxizao	SXZ	129	Zhenong 113	ZN113
60	Hanlv	HL	130	Yingchun	YC
61	Huangshagaofeng	HSGF	131	Cuifeng	CF
62	Mamulancha	MMLC	132	Baimaohou	BMH

63	Wuzao 1	WZ1	133	Soubei	SB
64	Dayewulong	DYWL	134	Qingxinqilan	QXQL
65	Fuyun 911	FY911	135	Jinji	JJ
66	Yinsun	YS	136	Zhenong12	ZN12
67	Ningzhou 7	NZ7	137	Zhuzhichun	ZZC
68	Wucha 6	WC6	138	Shuigucha	SGC
69	Ningzhouchangye	NZCY	139	Fuyun 20	FY20
70	Honghuapipa	HHPP	140	Jinshi	JS

Table S3. Turkey test and Dunn-Sidak test of the radical scavenging abilities of TPSs from different origins.

**Analysis items**	**Observations**	**MD**	**SEM**	**Turkey test**		**Sidak test**	
				***q* value**	***p* value**	***t* value**	***p* value**
scavenging ability of ·OH	Jiangxi-Hubei	0.2383	1.6628	0.2027	0.9888	0.1433	0.9986
	Yunnan-Hubei	3.3033		2.8094	0.1659	1.9866	0.2087
	Yunnan-Jiangxi	3.0650		2.6067	0.2054	1.8432	0.259
scavenging ability of O2-·	Jiangxi-Hubei	-0.9133	2.0253	0.6377	0.8951	-0.4509	0.9613
	Yunnan-Hubei	-1.8017		1.258	0.6587	-0.8895	0.7781
	Yunnan-Jiangxi	-0.8883		0.6203	0.9004	-0.4386	0.9642

MD: mean difference; SEM: standard error of the mean. Significant difference exits when $p < 0.05$.
